# Quantification of brominated flame retardants in soil

**DOI:** 10.2478/jvetres-2025-0070

**Published:** 2025-12-10

**Authors:** Wojciech Jerzy Pietroń, Monika Baran, Marek Pajurek, Szczepan Mikołajczyk

**Affiliations:** Department of Chemical Research of Food and Feed, National Veterinary Research Institute, 24-100 Puławy, Poland

**Keywords:** brominated flame retardants, method development, nBFRs, PBDEs, soil contamination

## Abstract

**Introduction:**

Soil quality plays a crucial role for farm animals, particularly those raised under free-range or organic conditions. Substances contaminating soil with a significant impact on food of animal origin are persistent organic pollutants (POPs). Recently, polybrominated diphenyl ethers (PBDEs) were included in this group. Novel brominated flame retardants (nBFRs) may also be included soon because they behave similarly in the environment. The aim of the study was to develop and validate a multicomponent method for determining 10 PBDE congeners and 8 compounds classified as nBFRs in soil.

**Material and Methods:**

Three soil samples were taken from potentially contaminated sites and three from theoretically uncontaminated sites. A high-resolution gas chromatography coupled with high-resolution mass spectrometry method was adapted.

**Results:**

The method demonstrated high sensitivity, precision and repeatability. The validated procedure enables quantification of ∑PBDEs in the range of 0.16–1700 ng·g^−1^ dry weight (d.w.) and ∑nBFRs in the range of 0.072–1130 ng·g^−1^ d.w. The optimised extraction and clean-up steps addressed the physicochemical diversity of the analytes and ensured reliable separation from co-contaminants. The levels of PBDEs in contaminated samples ranged from 0.23 to 485.7 ng·g^−1^ d.w., while nBFRs were detected at significantly lower levels (0.11–0.81 ng·g^−1^ d.w.).

**Conclusion:**

Given the absence of regulatory limits for BFRs in food and feed, and their documented presence in agricultural products, the developed method provides a valuable tool for environmental monitoring and risk assessment related to soil contamination and its potential impact on food safety.

## Introduction

Environmental pollution by persistent organic pollutants (POPs) always causes concern for animal health and food chain safety (4). Currently, 45 substances or commercial substance mixtures are included on the list of POPs, notable pollutants being - dibenzodioxins and furans (PCDD/Fs), polychlorinated biphenyls (PCBs) or perfluorooctanoic compounds (PFOS/PFOAs). Degradation of all of them in soil is extremely slow, taking from decades to centuries (38). They can be ingested by animals with soil during grazing or pecking, or absorbed by earthworms; therefore, soil is considered to be a significant source of animal exposure (8, 21). Consequently, soil analysis is an integral part of the source investigation for potential food contamination by PCDD/Fs and PCBs (34). Their physio-chemical properties lead to their accumulation in animal fat tissue and biomagnification in the food chain (39).

An important group of environmental contaminants and POPs are brominated flame retardants (BFRs). They are omnipresent in the environment because consumer protection standards set fire-resistance requirements for all materials, especially those of which the main components are flammable polymers (15). One of the easiest ways of increasing plastics’ resistance to combustion is modification of their composition by adding BFRs. Those substances include many chemical compounds, but so far, the most commonly used have been polybrominated diphenyl ethers (PBDEs), which have been added to plastics for over 30 years (2, 25, 30). These compounds are not tightly bound to polymer molecules, which is why they are systematically released from them into the environment. Over the past 20 years, it has been proven that chronic exposure to these compounds has negative health effects on humans and animals (3, 10). The compounds have a toxic effect on the liver, thyroid hormones and reproductive, nervous and immune systems, as well as on the metabolism of lipids and sugars (1, 18, 44). In animal studies, the most critical outcomes include neurodevelopmental effects on behaviour as well as reproductive and developmental toxicity (7). Additionally, the recent literature suggests that elevated PBDE concentrations may be linked to increased incidence of liver and breast cancers in humans and papillary thyroid cancers in rodents (6, 11, 12, 41). Nevertheless, previous reports on their carcinogenicity are inconsistent (7). It is noteworthy that in 2020, the International Agency for Research on Cancer gave high priority to assessing the potential carcinogenicity of the commercial penta-BDE mixture (13). Because of PBDEs’ durability in the environment and toxic properties, they have been classified as POPs under the Stockholm Convention, and their production has been banned (4). This led to their rapid replacement by alternative compounds, new or emerging brominated flame retardants (nBFRs), which have similar properties to PBDEs (31, 48). Global production of nBFRs is growing consistently and in 2019 was estimated at around 400,000 tonnes per year. The problem with the presence of BFRs does not end when a product containing them is disposed of, because together with the material, they end up in landfills (20, 23). One material into which these compounds are deposited among is sewage sludge, which is used to fertilise fields and can lead to soil contamination (36). In addition, soil can be contaminated with BFRs directly released from materials or as a result of atmospheric deposition of contaminated dust or ash (16, 47). Brominated flame retardants are persistent in the environment and can be ingested by terrestrial animals living in contaminated areas (5, 16, 22). In this way, they could enter into the chain of food of animal origin. The presence of these compounds in soil not only contributes to the exposure of animals and humans to them but could also have a harmful effect on soil microflora and basic chemical, physical and biological processes occurring in the soil (14, 35, 45).

The aim of the study was to develop and validate a multicomponent method for determining 10 PBDE congeners and 8 compounds classified as nBFRs in soil. The research procedure was based on the original technique of isotope dilution mass spectrometry. High-resolution gas chromatography coupled with high-resolution mass spectrometry (HRGC-HRMS) was used in the studies.

## Material and Methods

### Samples

To evaluate the method’s fitness for purpose and the contamination levels in soil from common land uses, six samples were obtained representing various contamination histories: from an open waste burning site, from a location known to have an ash burden, from a chicken run, from an orchard, from arable land and from a raspberry plantation. The first three soil samples were taken from the surface, at a depth of up to 3 cm, in the expectation of collecting substantially contaminated soil. The sampling depth on land under cultivation was 40 cm, and was specified in order to obtain soil contaminated at a level as low as possible, especially to sample soil free of potential atmospheric deposition of burning ash, which may contain BFRs.

### Analytes of interest

Standard solutions for 10 PBDE congeners and 8 nBFRs were used along with their isotopically labelled homologues. The only exceptions were BDE-49 and decabromodiphenyl ethane, as labelled equivalents were not commercially available when the analysis was conducted. Standard solutions of PBDE, hexabromobenzene, pentabromoethylbenzene, 2-ethylhexyl-2,3,4,5-tetrabromobenzoate, 1,2-bis(2,4,6-tribromophenoxy)ethane, bis(2-ethylhexyl) tetrabromophthalate, decabromodiphenyl ethane and ^13^C_6_ hexabromobenzene came from Cambridge Isotope Laboratories (Tewksbury, MA, USA). Standard solutions of TBX and PBT were delivered by Tokyo Chemical Industry (Tokyo, Japan) and Merck (Darmstadt, Germany). Isotopically labelled TBX and PBT came from Toronto Research Chemicals (Toronto, ON, Canada), and the labelled 2-ethylhexyl-2,3,4,5-tetrabromobenzoate, 1,2-bis(2,4,6-tribromophenoxy)ethane and bis(2-ethylhexyl) tetrabromophthalate were delivered by Wellington Laboratories (Guelph, ON, Canada). The standard solutions were prepared in isooctane, toluene and n-nonane. Table 1 provides a detailed list of the analytes.

**Table 1.** List of novel brominated flame retardants (nBFRs) and polybrominated diphenyl ethers (PBDEs) determined in soil using the developed method

	Name	CAS No.	Structural formula	Acronym	m/z
nBFR	2,3,5,6-Tetrabromo-p-xylene	23488-38-2	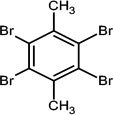	TBX	419.7182 421.7162
	2,3,4,5,6-Pentabromotoluene	87-83-2	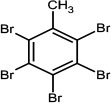	PBT	491.6307 499.6282
	2,3,4,5,6-Pentabromoethylbenzene	85-22-3	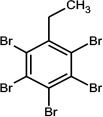	PBEB	485.6105 487.6085
	Hexabromobenzene	87-82-1	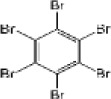	HBB	547.5074 549.5054
	2-Ethylhexyl 2,3,4,5-tetrabromobenzoate	183658-27-7	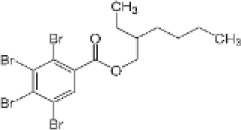	EH-TBB	418.6735 420.6714
	1,2-Bis(2,4,6-tribromophenoxy)ethane	37853-59-1	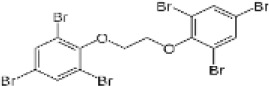	BTBPE	356.7948 358.7928
	Bis(2-ethylhexyl) tetrabromophthalate	26040-51-7	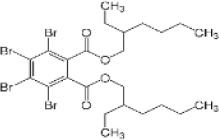	BEH-TBPH	462.6633 464.6613
	1,2-Bis(2,3,4,5,6-pentabromophenyl)ethane	84852-53-9	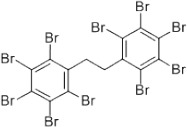	DBDPE	484.6032 486.6000
	2,4,4′-2,4,4′-Tribromodiphenylether	41318-75-6	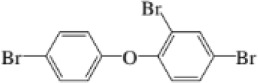	BDE-28	405.8027 407.8002
PBDE	2,2′,4,4′-Tetrabromodiphenyl ether	5436-43-1	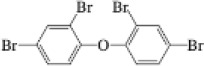	BDE-47	483.7132 485.7111
	2,2′,4,5′-Tetrabromodiphenyl ether	243982-82-3	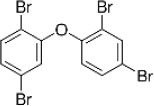	BDE-49	483.7132 485.7111
	2,2′,4,4′,5-Pentabromodiphenyl ether	60348-60-9	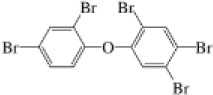	BDE-99	492.9691 563.6216
	2,2′,4,4′,6-Pentabromodiphenyl ether	189084-64-8	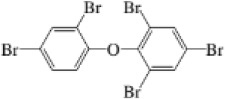	BDE-100	492.9691 563.6216
	2,2,3,4,4,5-Hexabromodiphenyl ether	67888-98-6	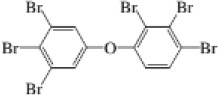	BDE-138	481.6980 483.6960
	2,2′,4,4′,5,5′-Hexabromodiphenyl ether	68631-49-2	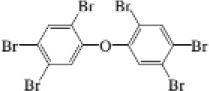	BDE-153	481.6980 483.6960
	2,2′,4,4′,5,6′-Hexabromodiphenyl ether	207122-15-4	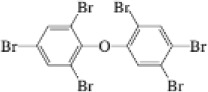	BDE-154	481.6980 483.6960
	2,2′,3,4,4′,5′,6-Heptabromodiphenyl ether	207122-16-5	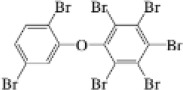	BDE-183	561.6060 563.6040
	Decabromodiphenyl ether	1163-19-5	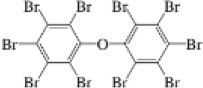	BDE-209	799.3330 801.3310

### Reagents and chemicals

Copper powder (<75 μm) was purchased from Sigma-Aldrich (St. Louis, MO, USA). Supelco aluminium oxide 150 acidic, Brockmann I, 37% HCl, dichloromethane, n-hexane (Chemsolute) and sulfuric acid (VI) were supplied by Merck. Silica gel was delivered by Fluka (Buchs, Switzerland). Florisil (100–200 mesh) was provided by LGC Standards (Wesel, Germany). Diatomaceous earth was sourced from Restek (Bellefonte, PA, USA). Helium (99.9999%) and nitrogen (99.999%) gases were delivered by Messer (Gumpoldskirchen, Austria). Freshwater sediment ERM-CC537a was purchased from the EU Directorate General Joint Research Center (Geel, Belgium).

### Sample preparation

About 500 g of each sample was dried at 80°C for 48 h in a UM 200 oven (Memmert, Schwabach, Germany). The dried samples were sieved through a mesh size of 1 mm and next through 0.5 mm. The samples obtained in this way were homogenised using a Turbula 3D Shaker Mixer (WAB, Muttenz, Switzerland). The moisture content was subsequently checked using an MA45C moisture analyser (Sartorius, Göttingen, Germany). If the moisture content exceeded 1%, the entire sample was dried again.

### Extraction and purification

A 10 g mass of sample was mixed with 0.2 g of powdered copper previously activated using 37% HCl and diatomaceous earth. Then the mixture was quantitatively transferred to the extraction cell, and isotopically labelled quantitative standards (^13^C and/or ^2^D) were added. The analytes were extracted with dichloromethane : n-hexane (1 : 1 v/v) using accelerated solvent extraction (Dionex, Sunnyvale, CA, USA) at 100 bar and 100°C. The extract was concentrated and transferred to a 50 mL centrifuge tube, topped up to 30 mL with dichloromethane : n-hexane, and 8 mL of concentrated H_2_SO_4_ was added. The mixture was shaken for 20 min and centrifuged, and the supernatant was transferred to a second tube and sulfuric acid (VI) was added again. The supernatant was then evaporated, and the residue was dissolved in 3 mL of n-hexane, purified and fractionated using normal-phase liquid chromatography. The obtained solution was applied to a column containing 4.4 g of 70–230 mesh silica gel impregnated with 44% H_2_SO_4_ (Supelco, now part of Merck). Some of the analytes were eluted with 20 mL of n-hexane and directly applied to a column containing 3 g of activated acidic alumina (150 mesh, Brockmann I, Merck). The analytes were eluted from the alumina with 20 mL of dichloromethane : n-hexane (50 : 50 v/v) mixture and 10 mL of dichloromethane to a 100 mL round-bottom F1 flask. The remaining analytes were eluted from the silica column with 25 mL of dichloromethane. The eluate was evaporated to dryness, dissolved in n-hexane and then purified using a column containing 1 g of deactivated Florisil (LGC). A round-bottom F1 flask was placed under the column, and then the analytes were eluted with 25 mL of dichloromethane. The obtained eluate was concentrated; the ^13^C_12_ BDE-139 recovery standard was added and the solution was evaporated to dryness under a gentle stream of nitrogen. The residue was dissolved in 100 μL of toluene, and then 1 μL was analysed using a DFS magnetic sector gas-chromatography high-resolution mass spectrometer (Thermo Fisher Scientific, Waltham, MA, USA).

### Instrumental analysis

The analytes were separated using an RTX-1614 15m × 0.25 mm × 0.1 μm capillary chromatography column (Restek). Instrumental analyses were performed separately for PBDE and nBFR using different temperature gradients. The PBDE and nBFR samples were injected using a programmable-temperature vaporisation injector. Its starting temperature was set at 120°C and the sample was injected in the splitless mode for 2 min, then the injector temperature was increased by 5°C·s^−1^ to 315°C and held for 18 min. The chromatograph oven temperature programme for PBDE determination was as follows: isothermal at 60°C for 1 min, 30°C/min to 220°C held for 3 min, 8°C/min to 240°C held for 2 min, 30°C/min to 300°C, and 2.5°C/min to 320°C held for 12.5 min. The helium flow rate was a constant 1.2 mL/min and the transfer line was maintained at 290°C. The chromatograph oven temperature programme for nBFRs determination was as follows: isothermal at 60°C for 1 min, 30°C/min to 200°C, 3.1°C/min to 210°C held for 2 min, 12°C/min to 240°C held for 1 min, 30°C/min to 275°C held for 2 min and 30°C/min to 315°C held for 5 min. The helium flow rate was once again a constant 1.2 mL/min and the transfer line was maintained at 315°C.

The mass spectrometer was calibrated by constant injection of the mass reference compounds: per-fluorotributylamine (FC-43) for nBFRs and perfluorokerosene (PFK) for PBDEs. The ion-source temperature was maintained at 280°C. The ionisation energy was 47.5 eV and the electron current was 1.0 mA. The quantifications were performed using multiple ion detection at a resolution equal or superior to 10,000 at 10% peak height.

### Quality assurance and quality control

Before routine analysis, the purity of all sorbents and solvents was verified. Throughout the chromatographic process, mass spectrometry resolution was consistently maintained at 10,000. Five standard concentrations were used to calibrate the HRGC-HRMS system. A blank sample and ERM-CC537 freshwater sediment were analysed with every sample series, which was of 12 samples or fewer. The recovery of ^13^C_12_ PBDE met US EPA 1614 method criteria requirements: 25–150% for BDE-28 to BDE-183, and 20–200% for BDE-209 (42). The recovery of the isotopically labelled nBFRs was in the acceptable range 20–150%. Solvent blank samples were added to each samples series and examined in the same way as matrix samples to control ingredient purity, within-lab interference and potential cross-contamination.

### Validation method

A validation study was performed in terms of the instrumental limit of quantification (iLOQ), limit of quantification (LOQ), limit of detection (LOD), specificity, linearity, accuracy (recovery) and precision (repeatability and within-laboratory reproducibility). These were determined using a blank soil sample spiked at a level near the LOD of the method. Ten samples prepared in the same way were spiked and analysed in two series. The LOD and LOQ were calculated based on standard deviation (SD) multiplied by 3 and 10, respectively. Calibration curves were constructed based on a standard solution with constant labelled analogues and six different concentrations of analytes. The linearity of the method was evaluated by plotting curves from spiked samples at four concentration levels. A comparison of the results of six replicates prepared on the same day at three different concentrations of PBDEs and nBFRs served as the assessment of repeatability. Table 2 shows the PBDE concentrations and Table 3 those of nBFRs. Within-laboratory reproducibility of the procedure was estimated by comparing the results when the same sample was prepared and analysed on three different days. Coefficients of variation were calculated for each analyte concentration level, and the analyte concentrations were calculated based on the native-to-labelled homologue ratio; therefore, their concentrations were not corrected for recovery. The procedure trueness was calculated by the formula (measured level/fortified level) × 100%. The relative standard uncertainty was calculated as the square root of repeatability, within-laboratory reproducibility, and the uncertainty of the certified reference material. The expanded uncertainty (U) was calculated as the ratio of the coverage factor (k = 2).

**Table 2.** Validation results of the developed method for polybrominated diphenyl ether quantification in soil

	BDE-28	BDE-47	BDE-49	BDE-99	BDE-100	BDE-138	BDE-153	BDE-154	BDE-183	BDE-209
LOD (ng/g d.m.)	0.0005	0.005	0.003	0.002	0.001	0.0006	0.0006	0.002	0.003	0.05
LOQ (ng/g d.m.)	0.002	0.016	0.009	0.006	0.004	0.002	0.002	0.005	0.011	0.15
Level 1										
Fortification (ng/g d.m.)	2.0	2.0	2.0	2.0	2.0	3.0	3.0	3.0	3.0	10.0
Repeatability										
Trueness (%)	10	8	1	11	11	8	6	9	12	5
CV (%)	4.3	7.0	6.4	7.7	7.4	5.7	6.1	4.9	8.2	3.8
Reproducibility										
Trueness (%)	6.0	3.6	–2.2	4.4	5.1	4.3	2.4	4.5	5.9	4.9
CV (%)	5.1	6.5	5.7	8.7	8.1	5.7	5.9	5.6	8.6	3.1
Uncertainty (%)	64	25	21	27	28	26	25	22	59	32
Level 2										
Fortification (ng/g d.m.)	4.0	4.0	4.0	4.0	4.0	6.0	6.0	6.0	6.0	20
Trueness (%)	5	4	2	6	7	6	3	6	9	2
CV (%)	4.1	5.5	8.8	4.5	4.0	2.1	2.8	1.6	6.0	6.8
Uncertainty (%)	64	23	29	22	24	15	21	19	57	34
Level 3										
Fortification (ng/g d.m.)	100	100	100	100	100	150	150	150	150	500
Trueness (%)	–3	–14	–18	–14	–13	–12	–13	–11	–14	–11
CV (%)	7.4	1.7	12.3	3.3	4.1	1.4	3.4	1.5	4.3	6.2
Uncertainty (%)	65	19	52	21	24	27	22	19	56	34
Linearity										
R^2^	1.0000	0.9994	0.9999	0.9999	0.9999	1.0000	1.0000	1.0000	0.9999	1.0000
β_1_	0.965	0.868	0.814	0.852	0.861	0.875	0.865	0.883	0.852	0.884
β_0_	0.196	–0.352	0.402	0.443	0.443	0.565	0.515	0.548	0.730	1.429

1 BDE – brominated diphenyl ether; LOD – limit of detection; LOQ – limit of quantification; Level 1/2/3 – enrichment levels; d.m. – dry matter; CV – coefficient of variation; R^2^ – coefficient of determination; β_0_ and β_1_ – are dimensional parameter vector in the equation y=β_0_ + β1x

**Table 3.** Validation results of the developed method for novel brominated flame retardant quantification in soil

	TBX	PBT	PBEB	HBB	EH-TBB	BTBPE	BEH-TBPH	DBDPE
LOD (ng/g d.m.)	0.0007	0.001	0.001	0.002	0.018	0.0014	0.005	0.005
LOQ (ng/g d.m.)	0.002	0.004	0.003	0.005	0.061	0.005	0.018	0.015
Level 1								
Fortification (ng/g d.m.)	0.050	0.050	0.20	0.050	0.20	0.20	0.50	1.0
Repeatability								
Trueness (%)	14	0	1	12	2	1	7	7
CV (%)	7.5	9.5	7.1	8.8	6.4	3.5	6.2	11.7
Reproducibility								
Trueness (%)	6.4	7.3	8.2	10.1	4.9	4.3	8.5	16.0
CV (%)	17	2	–5	18	1	3	3	0
Uncertainty (%)	18	30	23	39	22	13	27	40
Level 2								
Fortification (ng/g d.m.)	1.00	1.00	4.0	1.0	4.0	4.0	10.0	20
Trueness (%)	–4	–11	–7	3	–10	–5	–2	33
CV (%)	2.9	4.6	4.6	6.4	3.7	2.8	5.2	2.8
Uncertainty (%)	14	28	22	22	25	15	18	68
Level 3								
Fortification (ng/g. d.m.)	25	25	100	25	100	100	250	505
Trueness (%)	–7	–9	–18	–2	5	–9	–11	–22
CV (%)	5.2	6.6	3.3	4.9	6.4	5.2	8.6	19.6
Uncertainty (%)	23	28	40	17	24	25	36	71
Linearity								
R^2^	1.00000	1.00000	0.99997	1.00000	0.99997	1.00000	0.99998	0.99928
β_1_	0.927	0.911	0.817	0.974	1.047	0.912	0.885	0.776
β_0_	0.014	–0.005	0.158	0.022	–0.187	0.062	0.343	3.710

1 TBX – 2,3,5,6-tetrabromo-p-xylene; PBT – 2,3,4,5,6-pentabromotoluene; PBEB – 2,3,4,5,6-pentabromoethylbenzene; HBB – hexabromobenzene; EH-TBB – 2-ethylhexyl 2,3,4,5-tetrabromobenzoate; BTBPE – 1,2-bis(2,4,6-tribromophenoxy)ethane; BEH-TBPH – bis(2-ethylhexyl) tetrabromophthalate; DBDPE – 1,2-bis(2,3,4,5,6-pentabromophenyl)ethane; LOD – limit of detection; LOQ – limit of quantification; Level 1/2/3 – enrichment levels; d.m. – dry matter; CV – coefficient of variation; R^2^ – coefficient of determination; β_0_ and β_1_ – are dimensional parameter vector in the equation y=β_0_ + β1x

## Results

### Validation results

After initial level evaluation, a soil sample collected from a depth of 40 cm was selected for method validation. The LOQs for individual PBDE congeners ranged from 0.002 to 0.15 ng g^−1^ dry weight (d.w.), and for nBFRs from 0.002 to 0.061 ng g^−1^ d.w. The method’s trueness was determined, which, assaying fortified (spiked) samples, ranged from -18% to +12% and –12% to +7% for PBDE congeners and their sum, respectively (Tables 2 and 4). For nBFRs and their sum, it ranged from –15% to +33% and –15% to +12%, respectively (Tables 3 and 4). Determination of the test procedure accuracy (n = 6) from the reference material showed it to range from –8% to +33% for individual PBDE congeners. The variation under repeatability conditions ranged from 1.4% to 12% and from 2.8% to 20.0% for PBDE congeners and nBFRs, respectively. Individual compound groups’ sum contents were compared and in no case had variability exceeding 9% (Table 4). The coefficient of variation under reproducibility conditions ranged from –2.2% to 8.7% for PBDEs and from 4.3% to 16.0% for nBFRs, and did not exceed 8% for the sum of the tested compounds. The mean recoveries of labelled analogues ranged from 38.3% to 116.0% for PBDEs and from 41.5 to 112.0% for nBFRs. A range from 14 to 65% for PBDE congeners and one from 13 to 71% for nBFRs indicated the estimated expanded uncertainty (k = 2) using reference material or analyte-enhanced samples. Validation confirmed that the developed test method was suitable for its intended use. It allowed the analysis of soils for the content of 10 PBDE congeners and 8 nBFRs and their sums. The analytical procedure facilitated analyte quantification in the range from 0.16 to 1,700 ng·g^−1^ d.w. for ∑PBDEs and 0.072 to 1,130 ng·g^−1^ d.w. for ∑nBFRs.

**Table 4.** Validation results of the developed procedure for PBDEs’ (polybrominated diphenyl ethers) and nBFRs’ (novel brominated flame retardants sums in soil

	∑PBDE without BDE-209	∑PBDE	∑nBFR without DBDPE	∑nBFR	∑BFR
LOD (ng/g. d.m.)	0.014	0.049	0.022	0.022	0.057
LOQ (ng/g d.m.)	0.045	0.162	0.072	0.072	0.192
Level 1					
Fortification (ng/g. d.m.)	24	34	1.3	2.3	36
Repeatability					
Trueness (%)	9	7	5	6	7
CV (%)	6.2	5.4	1.2	5.0	5.1
Reproducibility					
Trueness (%)	–1	1	3	2	1
CV (%)	0.7	0.9	3.6	7.9	5.1
Uncertainty (%)	27	23	12	21	23
Level 2					
Fortification (ng/g. d.m.)	48	68	25	45	113
Trueness (%)	5	4	–5	12	7
CV (%)	3.6	4.2	3.6	2.9	3.0
Uncertainty (%)	16	16	15	27	19
Level 3					
Fortification (ng/g. d.m.)	1200	1700	625	1130	2830
Trueness (%)	–13	–12	–9	–15	–13
CV (%)	0.9	2.0	4.5	8.8	2.6
Uncertainty (%)	26	26	23	40	28
R^2^	0.99995	0.99996	1.00000	0.99986	0.99993
β_0_	0.87	0.87	0.91	0.85	0.86
β_1_	4.58	6.01	0.41	4.12	10.24

1 ∑PBDE – sum of polybrominated diphenyl ethers; ∑nBFR – sum of novel brominated flame retardants; DBDPE – 1,2-Bis(2,3,4,5,6-pentabromophenyl)ethane; ∑BFR – sum of brominated flame retardants; LOD – limit of detection; LOQ – limit of quantification; Level 1/2/3 – enrichment levels; d.m. – dry matter; CV – coefficient of variation; R^2^ – coefficient of determination; β_0_ and β_1_ – are dimensional parameter vector in the equation y=β_0_ + β_1_x

### Levels in soil samples

Polybrominated diphenyl ether content ranged from 0.23 ng·g^−1^ dry matter (d.m.) to 485.7 ng·g^−1^ d.m. in the investigated soil samples. The concentration of nBFRs was at markedly lower levels and was found in the range of 0.11–0.81 ng·g^−1^ d.m. As expected, soil samples with limited exposure to atmospheric precipitation, *e.g*. soil collected from a depth of 40 cm or from ground under cover, were characterised by the lowest concentrations of BFRs.

## Discussion

The soil is a potential source of brominated flame retardants for farm animals, especially those raised free-range or organically (24, 27). There are no maximum permitted levels of BFRs in food or feed; however, according to the European Food Safety Authority, current exposure of European consumers to PBDEs raises a health concern (7). The widespread presence of PBDEs was previously reported in food in a range from 0.001 to 10 ng/g fresh weight (24, 27, 29, 32, 33) and in feed (26). The presence of nBFRs in animal feed ranged from 0.04 to 3.05 μg/kg in material with a moisture content of 12% (31). Those authors found 78% of feed samples to contain at least one PBDE and 91% to contain nBFRs. Their extensive presence in food and feed could be related to their presence in soil. Therefore, an appropriate and validated procedure for BFR determination in soil was needed. The levels of BFRs in food and feed reported in the cited research were higher than those found in the current study, which could be connected with bioconcentration of those contaminants in the food chain. Very low environmental levels required a highly sensitive method, and HRGC-HRMS was selected as the detection technique. Only a few papers described method optimisation and validation parameters for BFR quantification in soil, and all of them used GC combined with mass spectrometry. Previously described methods used GC-MS/MS (19, 20), GC-MS (37, 40) and GC combined with ion trap mass spectrometry (43). Most researchers used electron ionisation, but according to Thorenz *et al*. (40), negative chemical ionisation has up to 10-fold better sensitivity. To the best of our knowledge, there is no published method for simultaneous PBDE and nBFR quantification in soil using an HRGC-HRMS technique. Only one paper describing a method for quantification of PBDEs in environmental samples used the same technique (17), and its authors did not report the LOD and LOQ. Additionally the reference method US EPA 1614 applied this technique to quantification of PBDEs in soil (42). The LOD and LOQ obtained in the current study are similar to the method detection limit and minimum level referred to by the US EPA method. The literature about similar analytical procedures is scarce. Only one paper described simultaneous PBDEs and nBFRs determination using GC-MS/MS method of standard resolution (19), giving method detection limits ranging from 0.01–4.8 ng/g d.w. and method quantification limits between 0.03 and 16 ng/g d.w. Those values were several times higher than LOQ of the method developed in this study.

Efficient analyte extraction is one of the most important steps of analyte determination. The physicochemical properties of BFRs require use of non-polar or semi-polar solvents or their mixture for extraction. Therefore, most of the methods published used toluene, n-hexane, isooctane or a mixture of dichloromethane, acetone or ethyl acetate. Similarly, an n-hexane–dichloromethane (1 : 1 v/v) mixture was used in the current study. Most of the papers noted accelerated solvent extraction in their protocols, similar to the current study. Soxhlet extraction was also incorporated into PBDE extraction; however, it is a much more time-consuming technique (42, 43). Additionally, column extraction supported by ultrasonic cavitation was also used for taking PBDEs out of soil (37).

Increasing the sample amount for extraction is necessary to increase method sensitivity. However, this forces additional clean-up of the extract before instrumental analysis. Most of the papers reported use of columns filled with silica gel impregnated with H_2_SO_4_, Florisil or aluminium oxide. A consequent suboptimal outcome of their use is inadequate discrimination between analytes; a method commonly used did not separate PBDEs from polychlorinated biphenyls (25), and only one paper was a record of such separation (17). Incomplete separation leads to inaccurate quantification, as overlapping peaks interfere in detection. The separate quantification of nBFRs requires modification of clean-up because of different elution profiles. Sufficient separation and clean-up of the extract were not achieved using a procedure for PBDE quantification (28). Therefore, the modification of the clean-up was necessary. The column containing 50 g of silica gel was replaced by fat oxidation using concentrated H_2_SO_4_ in a centrifugation tube. Additionally, the soil samples required the use of silica gel impregnated with 2% of powdered copper to remove potential sulphur contamination. This change was intended to prevent the capillary chromatography column from quick degradation. The concentration of PBDE congeners could even exceed 7,500 ng/g in soil treated with sewage-sludge (9), and the reported concentration of BFRs in soil reached 1,000 ng/g d.w. worldwide (46). Samples with such differing loads test method limits differently. This wide range of concentration in environmental samples and the lack of maximum permitted levels require a method with a wide range of concentrations in its validation (up to 500 ng/g d.w.).

Our results demonstrated good precision and repeatability, with an accuracy comparable to or exceeding that of prior research. A high uncertainty for PBDE was identified, which stemmed from a significant contribution of uncertainty from the reference material.

## Conclusion

The developed analytical method based on HRGC-HRMS has proved to be highly sensitive and suitable for the simultaneous quantification of 10 PBDE congeners and 8 nBFRs in soil samples. It demonstrated satisfactory precision, repeatability and trueness across a wide concentration range, with limits of quantification comparable to or better than those reported in previous studies. The validation results confirmed the method’s robustness, with acceptable recovery rates and uncertainty estimates. The validated method fills a critical gap in environmental monitoring. It enables reliable assessment of soil contamination, which is essential for evaluating potential risks to food safety and animal health, particularly in organic and free-range farming systems.
